# Supplementary material for: Mapping the evolution of fertility support policies in China: A content and instrumental analysis
Source: PLoS One. 2025 Oct 9;20(10):e0332137. doi: 10.1371/journal.pone.0332137 (PMC12510515; doi:10.1371/journal.pone.0332137)
Supplement: S1 Appendix — (ZIP) [file pone.0332137.s001.zip › S1 Appendix. 226 original policy documents/105-全国人民代表大会常务委员会法制工作委员会关于十三届全国人大以来暨2022年备案审查工作情况的报告(FBM-CLI.1.5146833).doc]

**全国人民代表大会常务委员会法制工作委员会关于十三届全国人大以来暨2022年备案审查工作情况的报告**

制定机关： [全国人大常委会法制工作委员会](https://www.pkulaw.com/law/chl?Aggs.IssueDepartment=203&way=textBasic)

公布日期：2022.12.30

施行日期：2022.12.30

时效性： [现行有效](https://www.pkulaw.com/law/chl?Aggs.TimelinessDic=01&way=textBasic)

效力位阶： [工作文件](https://www.pkulaw.com/law/chl?Aggs.EffectivenessDic=XA0105&way=textBasic)

法规类别： [法规规章备案](https://www.pkulaw.com/law/chl?Aggs.Category=00404&way=textBasic)

专题分类： [依法治国](https://www.pkulaw.com/law/chl?Aggs.SpecialType=019&way=textBasic)

全国人民代表大会常务委员会法制工作委员会关于十三届全国人大以来暨2022年备案审查工作情况的报告
——2022年12月28日在第十三届全国人民代表大会常务委员会第三十八次会议上
全国人大常委会法制工作委员会主任 沈春耀

全国人民代表大会常务委员会：
　　现将十三届全国人大以来暨2022年备案审查工作情况报告如下，请审议。
　　对行政法规、监察法规、地方性法规、自治条例和单行条例、经济特区法规、司法解释、特别行政区本地法律等规范性文件开展备案审查，是[宪法](https://www.pkulaw.com/chl/7c7e81f43957c58bbdfb.html?way=textSlc)法律赋予全国人大常委会的一项重要职权，是全国人大常委会履行[宪法](https://www.pkulaw.com/chl/7c7e81f43957c58bbdfb.html?way=textSlc)法律监督职责的一项重要工作。我国[宪法](https://www.pkulaw.com/chl/7c7e81f43957c58bbdfb.html?way=textSlc)和[立法法](https://www.pkulaw.com/chl/9073d435178b9633bdfb.html?way=textSlc)、监督法、地方组织法等法律都有关于备案审查方面的规定。党的十八大以来，以习近平同志为核心的党中央从推进全面依法治国、加强[宪法](https://www.pkulaw.com/chl/7c7e81f43957c58bbdfb.html?way=textSlc)法律实施和监督的战略高度，就加强备案审查工作作出一系列决策部署，党的十八届三中全会和四中全会、十九届二中全会、中央全面依法治国工作会议、中央人大工作会议和党中央有关文件都提出了明确的任务要求。十三届全国人大以来，每年常委会的工作报告、工作要点和有关工作计划都对备案审查工作作出部署、提出要求，常委会领导就做好备案审查工作多次作出指示批示，常委会会议每年都听取和审议法制工作委员会关于备案审查工作情况的报告。经过持续努力和有力推动，备案审查工作全面开展、扎实推进，逐步实现显性化、制度化、常态化，不断取得新进展新成效。围绕贯彻“有件必备、有备必审、有错必纠”的总要求，扩大备案范围，加大审查力度，丰富审查方式，增强纠错刚性，工作质量不断提升，建立健全备案审查工作衔接联动机制，加强对[宪法](https://www.pkulaw.com/chl/7c7e81f43957c58bbdfb.html?way=textSlc)法律实施情况的监督，依法纠正违反[宪法](https://www.pkulaw.com/chl/7c7e81f43957c58bbdfb.html?way=textSlc)法律、违背党中央精神或者不合时宜的规定。备案审查制度机制在保证党中央令行禁止、维护国家法治统一尊严权威、保护公民法人合法权利、发展全过程人民民主、促进社会公平正义、提升国家治理效能等方面的作用日益彰显。
　　**一、**十三届全国人大常委会备案审查工作取得新进展新成效
　　（一）实现“有件必备”。五年来，全国人大常委会接收报送备案的行政法规、监察法规、地方性法规、自治条例和单行条例、经济特区法规、司法解释以及香港、澳门两个特别行政区本地法律共7261件，其中，行政法规157件，监察法规1件，省级地方性法规2935件，设区的市级地方性法规2977件，自治条例和单行条例372件，经济特区法规242件，司法解释346件，香港、澳门特别行政区法律231件。全国人大常委会通过有关法律和决定，及时将国家监察委员会制定的监察法规和新类型地方性法规（即海南自由贸易港法规、浦东新区法规）纳入备案审查的范围。实行电子报备，规范报备方式，统一报备要求，提高报备效率。备案是审查的前提。总的看，规范性文件制定机关能够依法、及时将制定的规范性文件向全国人大常委会报送备案，自觉接受人大监督，“有件必备”的目标要求已经实现。
　　关于向人大常委会报送备案的规范性文件范围，法工委经研究认为，除了法律已有明确规定的以外，只要规范性文件的制定机关属于人大监督对象，其制定的规范性文件都应当纳入人大常委会备案审查范围；也就是说，“规范性文件在哪里，备案审查就跟到哪里”。近年来，我们推动地方人大常委会将本级“一府一委两院”制定的规范性文件纳入本级人大常委会备案审查范围；支持最高人民法院建立司法规范性文件备案审查制度机制。
　　（二）积极推进“有备必审”。根据有关法律的规定，国务院、中央军事委员会、最高人民法院、最高人民检察院和省、自治区、直辖市的人大常委会认为有关法规、司法解释等规范性文件同[宪法](https://www.pkulaw.com/chl/7c7e81f43957c58bbdfb.html?way=textSlc)或者法律相抵触的，可以向全国人大常委会书面提出进行审查的要求，由常委会工作机构送有关专门委员会进行审查、提出意见；有关国家机关和社会团体、企业事业组织以及公民认为有关法规、司法解释等规范性文件同[宪法](https://www.pkulaw.com/chl/7c7e81f43957c58bbdfb.html?way=textSlc)或者法律相抵触的，可以向全国人大常委会书面提出审查建议，由常委会工作机构进行研究，必要时送有关专门委员会进行审查、提出意见；有关专门委员会和常委会工作机构可以对报送备案的规范性文件进行主动审查。
　　五年来，在全国人大常委会领导下，法工委加大审查工作力度，努力实现“有备必审”。按照有关法律规定和常委会备案审查工作制度要求，依法对报送备案的各类法规、司法解释、特别行政区本地法律开展主动审查。除个别情形外，没有收到过国家机关提出的审查要求。公民、组织提出审查建议17769件，其中2018年1229件，2019年226件，2020年5146件，2021年6339件，2022年4829件，我们都认真研究处理。围绕贯彻落实党中央重大决策部署和重要法律实施，对生态环境保护、[民法典](https://www.pkulaw.com/chl/aa00daaeb5a4fe4ebdfb.html?way=textSlc)、[长江保护法](https://www.pkulaw.com/chl/adf90c0b432f8858bdfb.html?way=textSlc)、[人口与计划生育法](https://www.pkulaw.com/chl/d560ce000fba464cbdfb.html?way=textSlc)、[行政处罚法](https://www.pkulaw.com/chl/6add260f579ab541bdfb.html?way=textSlc)、食品药品安全等20多个领域的规范性文件，组织有关方面和地方人大开展集中清理工作和专项审查工作。对其他备案审查工作机构通过衔接联动机制移送的500多件法规、司法解释认真进行审查研究，提出处理意见。将不属于全国人大常委会审查范围的2500多件审查建议，根据不同情况，分送中央办公厅、司法部、最高人民法院、最高人民检察院和省级人大常委会研究处理。总的看，对各类规范性文件，基本实现“有备必审”、“应审尽审”，做到备案审查监督全覆盖。
　　根据审查工作情况，总的看，国务院、国家监察委员会、最高人民法院、最高人民检察院和有立法权的地方人大及其常委会，适应全面依法治国新形势新要求，依照法定权限和程序认真开展行政法规、监察法规、地方性法规、司法解释立改废释工作和报送备案工作，自觉坚持与[宪法](https://www.pkulaw.com/chl/7c7e81f43957c58bbdfb.html?way=textSlc)法律等上位法规定保持一致，不断提高规范性文件质量和水平，在贯彻党和国家大政方针、有效实施[宪法](https://www.pkulaw.com/chl/7c7e81f43957c58bbdfb.html?way=textSlc)法律、推动相关领域工作、有针对性解决问题等方面发挥了重要作用。
　　香港、澳门两个特别行政区将各自立法机关制定的本地法律向全国人大常委会备案，是香港、澳门两个特别行政区基本法明确规定两个特别行政区须履行的法定职责。多年来，全国人大常委会开展备案审查工作的范围，一直包括香港、澳门两个特别行政区立法机关制定的本地法律。五年来，香港特别行政区报送备案的本地法律127件，澳门特别行政区报送备案的本地法律104件。经初步审查，没有发现两个基本法规定的需要将报备的有关法律发回的情形。
　　（三）按照“有错必纠”原则督促推动制定机关纠错改正。法工委承担备案审查具体工作。我们依法履职尽责，认真开展规范性文件备案审查工作。不论是在依申请审查中，还是在主动审查、集中清理和专项审查中，都发现有些规范性文件或者其中有的规定存在与上位法规定相抵触、不一致或者明显不适当、不合理等问题。对存在需要纠正改正问题的有关规定，我们及时提出审查研究意见，督促和推动有关制定机关进行修改完善或者适时废止。
　　综合分析产生问题的原因，主要有以下几种情形：有的是制定机关对上位法有关规定、对党中央有关精神理解不透、把握不准，在贯彻落实过程中出现一定偏差；有的是规范性文件制定时间较早，上位法已经进行了修改或者作出了新的规定，实际情况已经发生很大的变化了，而原有规定未能及时清理，没有及时进行修改完善、实现与时俱进；有的是有关规定现在实际上已经不再执行，但是没有及时对有关规范性文件予以废止或者作出修改；有的是涉及的问题较为复杂，一直有不同理解和认识，都有一定道理，尚难作出合法性、合理性判断；也有的是个别制定机关政治意识不强、法治观念淡薄，在制定和执行有关规范性文件过程中打“擦边球”，甚至有意“放水”。
　　我们坚持问题导向，对审查发现的问题进行认真研究，通过沟通协商、提出书面审查意见、发函、约谈督促等方式，督促推动制定机关纠正改正；需要全国人大常委会作出相关决定的，依法按程序办理。五年来，累计督促推动制定机关修改完善或者废止各类规范性文件约2.5万件。
　　（四）加强备案审查制度建设。备案审查制度体系不断健全。常委会会议每年听取审议法工委关于备案审查工作情况的报告，并逐步实现常态化、制度化。推动地方各级人大常委会建立听取审议备案审查工作情况报告的制度；修改地方组织法，将听取和审议备案审查工作情况报告列为地方各级人大常委会的一项法定职权。全国人大常委会目前正在审议[立法法](https://www.pkulaw.com/chl/9073d435178b9633bdfb.html?way=textSlc)修正草案，其中包括完善备案审查制度和工作方面的重要内容。委员长会议通过《[法规、司法解释备案审查工作办法](https://www.pkulaw.com/chl/fe86b1479330e044bdfb.html?way=textSlc)》，对[宪法](https://www.pkulaw.com/chl/7c7e81f43957c58bbdfb.html?way=textSlc)和有关法律关于备案审查的规定作出进一步细化。法工委不断总结实践经验，制定有关备案审查工作的规范性文件，规范备案审查工作流程；同时，推动有关地方人大常委会制定或者修改备案审查法规，建立健全地方人大常委会备案审查工作制度机制。
　　（五）加强备案审查能力建设。法工委积极适应“有件必备、有备必审、有错必纠”的要求，着力提高自身综合素质和能力，扎实拓展和深化备案审查工作。建成全国统一的备案审查信息平台，初步建成国家法律法规数据库，推动建设省级法规规章规范性文件数据库，为实现应备尽备提供支撑。丰富审查方式，完善审查标准，细化工作流程。畅通相互间转送接续的机制渠道。加强对地方人大备案审查工作的联系指导，举办培训班，开展工作案例交流，探索建立案例指导制度。加强备案审查工作机构之间的协同联动。成立备案审查专家委员会，开展备案审查理论研究，通过多种方式和途径加强备案审查宣传工作。

　　**二、**在备案审查工作中加强合宪性问题审查研究，切实维护国家法治统一
　　推进合宪性审查工作是党的十九大和十九届二中、四中全会提出的明确要求，是新形势下加强[宪法](https://www.pkulaw.com/chl/7c7e81f43957c58bbdfb.html?way=textSlc)法律实施和监督的重要举措。习近平总书记在党的十九届二中全会上的重要讲话中指出：“全国人大常委会的备案审查工作，当然就包括审查有关规范性文件是否存在不符合[宪法](https://www.pkulaw.com/chl/7c7e81f43957c58bbdfb.html?way=textSlc)规定、不符合[宪法](https://www.pkulaw.com/chl/7c7e81f43957c58bbdfb.html?way=textSlc)精神的内容，要加强和改进这方面的工作。”这就为加强备案审查工作指明重点方向、提出了更高要求。
　　加强对合宪性、涉宪性问题审查研究。我们在备案审查工作中自觉增强[宪法](https://www.pkulaw.com/chl/7c7e81f43957c58bbdfb.html?way=textSlc)意识，推进合宪性审查工作，督促和推动制定机关纠正改正有关规定，及时启动有关程序废止不合时宜的法律规定和相关制度，弘扬[宪法](https://www.pkulaw.com/chl/7c7e81f43957c58bbdfb.html?way=textSlc)精神，维护[宪法](https://www.pkulaw.com/chl/7c7e81f43957c58bbdfb.html?way=textSlc)权威和国家法治统一。举若干工作实例如下。
　　——2018年3月，有全国政协委员提出提案，建议对收容教育制度进行合宪性审查。法工委经认真研究提出，收容教育制度是1991年根据全国人大常委会有关决定和国务院有关行政法规实行的、对有关人员进行必要约束和管控的一项制度措施，制定程序和内容是有[宪法](https://www.pkulaw.com/chl/7c7e81f43957c58bbdfb.html?way=textSlc)法律依据的；执行20多年后再来审视，情况已经发生了很大变化；经调研沟通，有关方面逐步形成共识，继续执行有关制度措施已不合时宜；为深入贯彻全面依法治国精神，法工委建议有关方面适时提出议案，废止有关收容教育法律规定和相关制度。2019年12月，十三届全国人大常委会第十五次会议审议了国务院提出的有关议案，作出了《[全国人民代表大会常务委员会关于废止有关收容教育法律规定和制度的决定](https://www.pkulaw.com/chl/1ea53f89482c77b7bdfb.html?way=textSlc)》。
　　——有的司法解释规定，人身损害赔偿案件中，对城镇居民和农村居民分别以不同标准计算残疾赔偿金和死亡赔偿金。2020年有公民提出审查建议，认为计算标准不同会导致案件审理出现不公平现象，与[宪法](https://www.pkulaw.com/chl/7c7e81f43957c58bbdfb.html?way=textSlc)有关精神不一致。我们认真进行审查研究，认为随着经济社会发展和城乡融合发展，有关计算标准的差异应当逐步改变和取消；建议制定机关在总结试点经验基础上适时修改完善有关司法解释，统一城乡居民人身损害赔偿标准。有关司法解释经修改后自2022年5月1日起实施，残疾赔偿金、死亡赔偿金以及被扶养人生活费统一采用城镇居民标准计算。
　　——2021年，国务院有关主管部门对有的民族自治地方民族教育条例等法规提出合宪性审查建议，认为条例中的有关规定存在合宪性问题，不利于促进民族交往交流交融。我们经审查研究认为，[宪法](https://www.pkulaw.com/chl/7c7e81f43957c58bbdfb.html?way=textSlc)和[国家通用语言文字法](https://www.pkulaw.com/chl/42d5d52adf7567f0bdfb.html?way=textSlc)已经对推广普及国家通用语言文字作出了明确规定，2015年全国人大常委会对[教育法](https://www.pkulaw.com/chl/b50e1d673a3faddfbdfb.html?way=textSlc)有关规定作出重要修改，包括民族地区在内的全国各地区应当全面推行国家通用语言文字教育教学，有关地方性法规中的相关内容应予纠正。经与有关方面沟通，制定机关已经废止有关法规。
　　——有的地方性法规规定，有关行政部门为调查计划生育违法事实，可以要求当事人进行亲子鉴定；对拒不配合的，处以1万元以上5万元以下罚款。2021年，有公民对上述规定提出审查建议。我们经审查认为，亲子关系涉及公民人格尊严、身份、隐私和家庭关系和谐稳定，属于公民基本权益，受[宪法](https://www.pkulaw.com/chl/7c7e81f43957c58bbdfb.html?way=textSlc)法律保护，地方性法规不宜规定强制性亲子鉴定的内容，也不应对此设定相应的行政处罚、处分、处理措施。经沟通，制定机关已对相关规定作出修改。
　　加强对有争议问题的研究。例如，有司法解释规定，上级人民检察院可以依法统一调用辖区内的检察人员办理案件，经上级人民检察院作出调用决定，被调用的检察官可以代表办理案件的人民检察院履行出庭支持公诉等各项检察职责。有公民对此规定提出审查建议。对被调用的检察人员代表所调用的人民检察院履行出庭支持公诉等各项检察职责是否需要经本级人大常委会作出相关任职决定，实践中存在不同认识和做法，涉及对[人民检察院组织法](https://www.pkulaw.com/chl/76b118e6e1459250bdfb.html?way=textSlc)有关规定的理解。我们经研究认为，[宪法](https://www.pkulaw.com/chl/7c7e81f43957c58bbdfb.html?way=textSlc)和有关组织法等法律共同构成检察权行使的法律依据，根据[人民检察院组织法](https://www.pkulaw.com/chl/76b118e6e1459250bdfb.html?way=textSlc)有关规定，上级人民检察院可以调用辖区的检察人员办理案件；被调用的检察人员代表办理案件的人民检察院履行出庭支持公诉等各项检察职责的，须经本级人大常委会作出相关任职决定。我们已向有关制定机关提出了研究意见，建议予以考虑。
　　加强与有关机关协同联动。例如，有的省级法院、检察院、公安厅就办理袭警罪案件联合发文。我们经审查研究认为，该联合发文涉及犯罪构成具体内容，扩大了法定袭警罪、妨害公务罪的惩治范围，超出了[刑法](https://www.pkulaw.com/chl/39c1b78830b970eabdfb.html?way=textSlc)规定范围；[立法法](https://www.pkulaw.com/chl/9073d435178b9633bdfb.html?way=textSlc)是重要[宪法](https://www.pkulaw.com/chl/7c7e81f43957c58bbdfb.html?way=textSlc)性法律，有关联合发文不符合[立法法](https://www.pkulaw.com/chl/9073d435178b9633bdfb.html?way=textSlc)关于“两高”以外的审判机关、检察机关不得作出具体应用法律的解释的规定，超出了制定机关权限。我们通过衔接联动机制与有关方面沟通，多方面协同行动、督促推动，制定机关很快发文通知停止执行有关文件。最高人民法院、最高人民检察院针对这一事例要求有关法院、检察院开展司法规范性文件自查清理，重申地方一律不得制定在本辖区普遍适用的、涉及具体应用法律问题的司法解释性质文件，制定的其他规范性文件不得在法律文书中援引。

　　**三、**持续开展重点领域规范性文件集中清理和专项审查工作，推动党中央重大决策部署、国家重要法律和法治措施的贯彻落实
　　五年来，围绕推动党中央重大决策部署、国家重要法律和法治措施的贯彻落实，在全国人大常委会领导下，我们组织开展重点领域规范性文件集中清理和专项审查工作，积极推进国家各方面工作法治化。
　　比如，根据党中央关于全面加强生态环境保护坚决打好污染防治攻坚战的重大决策部署和[全国人大常委会关于全面加强生态环境保护依法推动打好污染防治攻坚战的决议](https://www.pkulaw.com/chl/eef47967dc6290c5bdfb.html?way=textSlc)，2018年针对生态环保领域、2020年针对野生动物保护领域、2021年针对长江流域保护领域开展规范性文件集中清理和专项审查工作。2022年以来，围绕持续深入打好蓝天、碧水、净土保卫战，对长江流域保护专项清理后续工作进行跟踪督促，继续加大涉及生态资源与环境保护、污染防治、城市绿化等方面法规审查力度，着重审查存在与[大气污染防治法](https://www.pkulaw.com/chl/56d189e827f94116bdfb.html?way=textSlc)、[水污染防治法](https://www.pkulaw.com/chl/029b95a88a65c9d6bdfb.html?way=textSlc)、[土壤污染防治法](https://www.pkulaw.com/chl/d1bcb8c5a78e6554bdfb.html?way=textSlc)、[固体废物污染环境防治法](https://www.pkulaw.com/chl/38e38019d3cd06adbdfb.html?way=textSlc)等法律规定不一致的问题。对审查中发现与上位法规定相抵触，特别是存在放松管控、降低标准等立法“放水”问题的，要求制定机关及时修改、废止；对滞后于新制定或者新修改的上位法规定的，要求制定机关及时作出相应的调整完善；对符合上位法立法目的，确立比上位法规定更高的保护标准、更严格的保护制度的，应当予以支持。通过持续开展集中清理工作和专项审查工作，推动党中央重大决策部署和相关领域法律制度的贯彻落实，为生态文明建设和美丽中国建设提供有力法治保障。
　　再如，[民法典](https://www.pkulaw.com/chl/aa00daaeb5a4fe4ebdfb.html?way=textSlc)出台后第二天，十九届中央政治局就切实实施[民法典](https://www.pkulaw.com/chl/aa00daaeb5a4fe4ebdfb.html?way=textSlc)进行集体学习，习近平总书记发表重要讲话，明确提出：“对同[民法典](https://www.pkulaw.com/chl/aa00daaeb5a4fe4ebdfb.html?way=textSlc)规定和原则不一致的国家有关规定，要抓紧清理，该修改的修改，该废止的废止。”贯彻习近平总书记重要指示要求，我们组织开展了[民法典](https://www.pkulaw.com/chl/aa00daaeb5a4fe4ebdfb.html?way=textSlc)涉及领域规范性文件集中清理工作和专项审查工作。2021年，对[民法典](https://www.pkulaw.com/chl/aa00daaeb5a4fe4ebdfb.html?way=textSlc)专项清理后续工作进行跟踪督促。今年以来，持续加强对[民法典](https://www.pkulaw.com/chl/aa00daaeb5a4fe4ebdfb.html?way=textSlc)涉及领域法规审查纠正力度，巩固和扩大专项清理成果。有的地方性法规规定，业主大会可以在管理规约、业主大会议事规则中，对拒付物业服务费、公共水电分摊费和不交存物业维修资金的业主参加业主大会、行使投票权等权利进行限制。我们经审查认为，不加区分地将业主拒付物业服务费、物业维修资金等不履行业主义务行为同业主行使共同管理权挂钩，进而限制业主的建筑物区分所有权，与[民法典](https://www.pkulaw.com/chl/aa00daaeb5a4fe4ebdfb.html?way=textSlc)有关规定不符合。有的地方性法规规定，热用户应当在每年采暖期开始前30日内交纳本采暖期的热费，不按时交纳的，供热单位可以对其暂缓供热、限制供热或者停止供热。我们经审查认为，热用户采暖费的交纳时间和逾期不交纳热费的法律责任，应当遵循[民法典](https://www.pkulaw.com/chl/aa00daaeb5a4fe4ebdfb.html?way=textSlc)有关规定。有的地方性法规规定，进行水力发电需要通过招标、拍卖等方式从政府取得“水能资源开发利用权”并缴纳出让金。我们经审查认为，地方自行规定的“水能资源开发利用权”，具有用益物权性质，不符合[民法典](https://www.pkulaw.com/chl/aa00daaeb5a4fe4ebdfb.html?way=textSlc)确立的物权法定原则。上述问题和类似一些问题，有的已经解决，有的正在解决。
　　又如，本届以来，根据党中央2015年关于“我国人口发展呈现出重大转折性变化”的重大判断精神、2020年关于“增强生育政策包容性”的精神、2021年关于“优化生育政策促进人口长期均衡发展”的重大决策部署精神，我们持续关注和加强人口与计划生育领域规范性文件审查工作，及时组织开展集中清理工作和专项审查工作，本届以来历次备案审查工作情况报告都有涉及计划生育方面的内容。2017年开始、2018年接续，推动规范性文件制定机关对企业职工“超生即辞退”的规定作出修改；2019年，推动制定机关对国家工作人员“超生即开除”的规定作出修改。法工委在2019年备案审查工作情况报告中提出，这类规定虽目前有上位法的一定依据，但总的看已经不适应、不符合党和国家关于改革完善计划生育服务管理的精神和方向，应予适时调整，建议有关方面研究启动修改完善工作。2021年8月，十三届全国人大常委会第三十次会议对[人口与计划生育法](https://www.pkulaw.com/chl/d560ce000fba464cbdfb.html?way=textSlc)作出重要修改。为确保修改后的[人口与计划生育法](https://www.pkulaw.com/chl/d560ce000fba464cbdfb.html?way=textSlc)正确实施，我们组织开展了涉及计划生育内容的法规、规章等规范性文件集中清理和专项审查工作，推动制定机关修改、废止了一批相关规范性文件；督促制定机关取消社会抚养费征收规定，废止计划生育相关处罚、处分规定，将入户、入学、入职等与个人生育情况全面脱钩；同时，建议有关方面开展涉及计划生育内容的党内法规、规范性文件专项清理，督促有关方面及时修改对享受生育保险待遇作出不适当限制的规定。
　　开展集中清理和专项审查工作，拓展了备案审查工作思路，增强了备案审查工作的针对性、系统性、时效性，有利于发挥法律体系整体功效和治理体系整体效能。实践表明，及时组织开展重点领域规范性文件集中清理和专项审查工作，对于实现相关领域上下联动、左右协同，保证党中央精神全面贯彻、一体遵循，保证国家重要法律和法治措施有效实施，促进法律体系内在科学和谐统一，推进国家治理体系和治理能力现代化，具有重要意义。

　　**四、**在备案审查工作中践行全过程人民民主重大理念，努力增强人民群众法治获得感
　　发展全过程人民民主，是新时代新征程做好备案审查工作必须遵循的重要原则、必须贯彻的重大理念。就备案审查工作来说，有两个方面的要求：一是通过备案审查工作，运用法治方式解决人民群众关心关注的实际问题，增强人民群众法治获得感；二是在备案审查工作中不断拓展和健全人民群众有序参与立法、表达意愿关切的途径和形式，丰富和发展全过程人民民主的生动实践。近年来，我们注重健全备案审查工作吸纳民意、汇聚民智的机制、渠道和方式，努力使备案审查制度机制和工作实践成为新时代新征程践行全过程人民民主重大理念的具体体现。
　　拓宽人民群众诉求表达的渠道和机制。[立法法](https://www.pkulaw.com/chl/9073d435178b9633bdfb.html?way=textSlc)、监督法等法律规定，公民、组织认为法规、司法解释等规范性文件同[宪法](https://www.pkulaw.com/chl/7c7e81f43957c58bbdfb.html?way=textSlc)或者法律相抵触的，可以向全国人大常委会书面提出进行审查的建议，由常委会工作机构进行研究。这是保证人民群众对国家立法工作知情权、参与权、表达权、监督权的制度设计和理念体现。2019年，在中国人大网开通审查建议在线提交平台，实现审查建议“一键提交”。三年来，我们共收到公民、组织网上提出的审查建议11800余件，约占五年来审查建议总数的66.4%。法工委每年都向常委会报告备案审查工作情况，并将年度备案审查工作情况报告通过中国人大网和全国人大常委会公报向社会公布。通过多种渠道和方式做好备案审查宣传工作，增强工作透明度，拓展群众参与度。把备案审查工作同办理代表议案建议、委员提案工作结合起来，同基层立法联系点工作结合起来，同立法修法工作结合起来，同联系指导地方人大备案审查工作结合起来，努力通过各种渠道和形式了解社情民意，努力通过备案审查工作回应社会关切，努力使备案审查制度和工作成为践行全过程人民民主重大理念的具体形式和制度载体。
　　认真研究处理收到和转来的审查建议。人民群众提出的每件审查建议，“两会”期间全国人大代表、全国政协委员提出的每件建议、议案、提案，我们都认真研究，督促纠正了一批人民群众集中反映、影响老百姓切身利益、直接涉及公民权利义务的法规、司法解释等规范性文件，保障全过程人民民主落到实处。五年来，共收到1.7万多件审查建议，审查建议人来自不同地域、行业、群体，包括工人、农民、个体工商户、专家学者、基层行政执法人员、律师、学生、人大代表、政协委员、企业事业单位、有关国家机关等；审查建议内容广泛，涉及诸多领域，或表达权益诉求，或关注法治进步，或反映群众急难愁盼问题，或呼吁解决制度衔接问题。各种意见反映和诉求表达，总的看属于建设性、正能量，是我国全过程人民民主的具体体现。我们对审查建议逐一登记、逐件研究；必要时，与审查建议人沟通，了解来自基层和各方面的真实声音，增强审查研究的针对性、实效性；提出处理意见后，通过书面发函、电话沟通等方式及时作出反馈。以研究处理审查建议为抓手，积极推动解决人民群众普遍关注的问题。比如，2021年，根据全国人大代表提出的建议，督促纠正个别地方以户籍限制从事出租车司机职业的问题；2022年，又进一步梳理研究、推动14个地方纠正了类似问题。
　　广泛听取意见，加强调研论证，做到集思广益。在审查研究工作中，对可能存在问题的规范性文件，充分听取制定机关的情况说明，通过座谈会、论证会、委托研究、走访调研等方式，听取专门委员会、国家机关、社会团体、企事业单位、人大代表、专家学者以及利益相关方的意见，从法治原则出发分析其中利弊得失。比如，2021年，有的地方性法规规定，停车人应当按照规定缴纳道路停车费用，逾期未缴纳的进行催缴同时并处200元以上1000元以下罚款。有公民对有关罚款规定提出审查建议。针对这一审查建议，我们请制定机关说明情况，请执法部门提供有关规定实施情况，向有关部门了解停车收费改革的相关政策，向审查建议人进一步询问提出审查建议的考虑，通过多种方式了解、走访居民127户，在此基础上与有关制定机关就法规修改方案反复交换意见，力求取得平衡和共识。制定机关后来对有关规定作出修改，调整完善罚款的额度和程序，并督促有关单位出台具体办法，处理结果得到社会各方面普遍认可。我们还注重借助“外脑”，建立备案审查专家委员会，围绕备案审查工作相关问题，组织专家进行论证；探索发挥智库作用，委托有关高校、研究机构开展专题研究。
　　积极参与建设国家法律法规数据库。备案审查信息平台建成后，法工委与常委会办公厅有关方面共同协作，初步建成国家法律法规数据库，全面收录我国现行有效的各类法律法规，为社会公众持续提供权威版本的法律法规等文件，提供看得见、找的着、用得上的法律产品，积极回应和满足人民群众的法治需求。

　　**五、**2022年备案审查工作总体情况
　　2022年，在全国人大常委会领导下，法工委与有关方面协同配合，对法规、司法解释和特别行政区本地法律依法开展备案审查工作，持续取得新进展。
　　一年来，全国人大常委会办公厅收到报送备案的法规、司法解释及特别行政区法律共1172件，其中行政法规23件，省、自治区、直辖市地方性法规500件，设区的市、自治州地方性法规495件，自治条例和单行条例68件，经济特区法规40件，司法解释20件，香港特别行政区法律11件，澳门特别行政区法律15件。制定《法规、司法解释形式审查工作程序》，加强形式审查，对存在报备问题的56件法规督促报备机关及时予以改正。
　　法工委对报送备案的法规、司法解释及特别行政区本地法律依职权主动进行审查。收到公民、组织提出的审查建议4829件，其中属于全国人大常委会审查范围的4067件，包括针对行政法规和国务院决定提出的108件，针对地方性法规、自治条例和单行条例、经济特区法规提出的3815件，针对司法解释提出的144件。没有收到有关国家机关提出的审查要求。对不属于全国人大常委会审查范围、可能存在问题的333件审查建议移送有关备案审查工作机构研究处理，其中移送中央办公厅3件、司法部62件、最高人民法院6件、最高人民检察院3件、省级地方人大常委会259件。研究处理其他备案审查工作机构移送或提出的审查建议286件。对2021年专项清理工作有关事项进行跟踪督促，组织开展物业管理、生育保险等领域规范性文件集中清理和专项审查工作。截至目前，共督促、推动制定机关修改、废止法规、司法解释及其他规范性文件781件。
　　落实修改后的地方组织法，推动地方各级人大常委会建立健全听取和审议备案审查工作情况报告制度。2017年12月，全国人大常委会会议首次听取和审议全国人大常委会法工委关于[十二届全国人大以来暨2017年备案审查工作情况的报告](https://www.pkulaw.com/chl/f815f47b91ee0da0bdfb.html?way=textSlc)，之后逐步实现常态化、制度化。近年来，为了增强备案审查工作整体实效，我们推动地方人大常委会根据实际情况建立健全相关制度机制。2019年，实现省级人大常委会听取审议备案审查工作情况报告制度全覆盖；2021年，实现设区的市级全覆盖；2022年，力争实现县级全覆盖。2022年3月，修改后的地方组织法对人大常委会听取审议备案审查工作情况报告制度已经作出明确规定。
　　为推动解决地方立法共性问题，统一审查标准和尺度，探索建立备案审查案例指导制度，选编有关事例实例作为备案审查工作案例印送地方人大参考。收集整理一些地方人大常委会审查纠正处理的典型事例139件，作为工作交流材料。召开全国省级法规规章规范性文件数据库建设工作推进会，总结交流浙江、广东、重庆、宁夏四地试点经验，全面启动省级法规规章规范性文件数据库建设工作。围绕现行[宪法](https://www.pkulaw.com/chl/7c7e81f43957c58bbdfb.html?way=textSlc)颁布施行40周年，深入推进备案审查理论研究。
　　2022年是十三届全国人大常委会依法履职、开展工作的第五年。法工委对过去五年备案审查工作进行了回顾总结，力求深化对备案审查工作功能、特点、规律的认识。备案审查工作情况除了在每年报告中已作反映外，五年工作的总体情况在本报告前述部分中已作了概述。五年来，备案审查工作取得新进展新成效，是多方面支持和推动的结果，也是我们自身持续努力和扎实工作的结果。作为直接从事和承担这项工作的机构，我们有切身的体会和感受。概括起来，主要有以下几点工作体会：一是必须坚持以习近平新时代中国特色社会主义思想为指导，深入贯彻习近平法治思想，始终围绕加强[宪法](https://www.pkulaw.com/chl/7c7e81f43957c58bbdfb.html?way=textSlc)实施和监督开展备案审查工作，确保备案审查工作正确政治方向；二是必须坚持党的全面领导，服务党和国家工作大局，全面贯彻党的路线方针政策和党中央决策部署，确保各类规范性文件与党中央精神保持一致；三是必须坚持以人民为中心，贯彻全过程人民民主重大理念和实践要求，保障人民群众通过备案审查制度依法提出意见、表达诉求，努力做到民有所呼、我有所应，切实维护人民根本利益；四是必须坚持全面依法治国，坚持[宪法](https://www.pkulaw.com/chl/7c7e81f43957c58bbdfb.html?way=textSlc)法律至上，全面推进国家各方面工作法治化，加强人权法治保障，维护和促进社会公平正义，坚决纠正违宪违法的规范性文件，切实维护[宪法](https://www.pkulaw.com/chl/7c7e81f43957c58bbdfb.html?way=textSlc)法律权威和国家法治统一；五是必须坚持中国特色社会主义政治制度自信，坚持和完善人民代表大会制度，充分发挥人民代表大会制度优势，实行正确监督、有效监督、依法监督，构建起覆盖各类规范性文件的备案审查监督体系，增强备案审查制度整体功效。这些体会深化了我们对备案审查工作规律性的认识，应当作为今后加强和改进工作的重要原则长期坚持并在实践中不断丰富发展。
　　同时，我们在回顾总结中也感到工作还有很多差距和不足，工作质量、水平、效率有待提高；对一些重点难点问题还缺乏深入研究，对合宪性合法性合理性问题在深刻理解、准确把握、妥善处理上还有差距；新形势新任务对备案审查工作提出了新的更高要求，我们不论从自身素质还是从工作机制上都还有不适应、不符合的问题；如何发挥人大和各方面积极性，如何加强理论研究和宣传工作等，这些都需要我们在未来工作中不断加强和改进。

　　**六、**2023年备案审查工作初步安排和考虑
　　2023年是贯彻落实党的二十大精神开局之年。党的二十大强调“加强[宪法](https://www.pkulaw.com/chl/7c7e81f43957c58bbdfb.html?way=textSlc)实施和监督、健全保证[宪法](https://www.pkulaw.com/chl/7c7e81f43957c58bbdfb.html?way=textSlc)全面实施的制度体系”，明确提出“完善和加强备案审查制度”。2023年备案审查工作总体要求是，全面贯彻党的二十大和二十届一中全会精神，深刻领悟“两个确立”的决定性意义，深入学习贯彻习近平新时代中国特色社会主义思想，加强备案审查工作，提升备案审查质量，完善备案审查制度，通过备案审查制度和工作助力全面依法治国，发展全过程人民民主，维护[宪法](https://www.pkulaw.com/chl/7c7e81f43957c58bbdfb.html?way=textSlc)法律权威和国家法治统一，发挥中国特色社会主义法律体系整体功效，为法治中国建设作出积极贡献。
　　一是加强[宪法](https://www.pkulaw.com/chl/7c7e81f43957c58bbdfb.html?way=textSlc)实施和监督。健全保证[宪法](https://www.pkulaw.com/chl/7c7e81f43957c58bbdfb.html?way=textSlc)全面实施的制度体系，完善推进合宪性审查工作的思路、机制和方式方法。加强对法规、司法解释等规范性文件的合宪性、涉宪性问题审查研究，在备案审查工作中准确把握和阐明与[宪法](https://www.pkulaw.com/chl/7c7e81f43957c58bbdfb.html?way=textSlc)规定有关的内容。认真研究、妥善处理规范性文件中合宪性、涉宪性问题，及时督促纠正改正与[宪法](https://www.pkulaw.com/chl/7c7e81f43957c58bbdfb.html?way=textSlc)规定、[宪法](https://www.pkulaw.com/chl/7c7e81f43957c58bbdfb.html?way=textSlc)原则、[宪法](https://www.pkulaw.com/chl/7c7e81f43957c58bbdfb.html?way=textSlc)精神不一致不相符的问题。
　　二是完善和加强备案审查制度。修改[立法法](https://www.pkulaw.com/chl/9073d435178b9633bdfb.html?way=textSlc)、监督法，研究出台关于完善和加强备案审查制度的决定，进一步健全备案审查制度体系。提高全面落实“有件必备、有备必审、有错必纠”的水平。围绕贯彻党中央重大决策部署和重要法律实施，加强主动审查、专项审查，建立健全重点领域法律法规等规范性文件集中清理常态化机制。依法对香港、澳门特别行政区法律开展备案审查。
　　三是着力提高备案审查工作质量。进一步完善备案审查工作机制，细化审查标准，规范审查流程，加强调研论证，强化审查说法说理，提升审查工作科学化水平。更好地发挥备案审查专家委员会作用。增强制度刚性，坚决纠正一切违宪违法的规范性文件，维护国家法治统一、尊严、权威。通过备案审查工作推动有关方面完善相关制度规定，支持有关地方开展地方立法先行先试探索。
　　四是坚持和发展全过程人民民主。不断畅通人民权益表达渠道和参与过程，认真做好代表、委员提出的或者公民、组织提出的审查建议的研究、处理和反馈工作，完善审查建议办理机制，提高重点、难点审查建议办理成效，提升审查工作民主含量和质量，把备案审查工作和制度建设成为人大民主民意重要表达平台和载体。
　　五是加强对地方人大备案审查工作的联系指导。适时召开人大备案审查工作座谈会。总结近年来的实践经验，结合有关法律法规修改，举办工作培训班。运用备案审查工作案例推动工作交流互鉴。健全听取和审议备案审查工作情况报告制度，提高报告质量，通过报告制度推动备案审查工作。推进省级法规规章规范性文件数据库建设。
　　六是加强备案审查理论研究和宣传工作。围绕有关法律修改，紧密结合当代中国法治实践，推动学界加强合宪性审查、备案审查理论研究，促进理论与实践融合发展。聚焦[宪法](https://www.pkulaw.com/chl/7c7e81f43957c58bbdfb.html?way=textSlc)实施和监督，探讨中国特色[宪法](https://www.pkulaw.com/chl/7c7e81f43957c58bbdfb.html?way=textSlc)实施和监督理论体系、话语体系。创新备案审查宣传工作机制，提高宣传效果，讲好中国备案审查故事。

#### 本篇引用的法规

##### 中央法规

[中华人民共和国人口与计划生育法(2021修正)](https://www.pkulaw.com/chl/d560ce000fba464cbdfb.html?way=textRightFblx)

[中华人民共和国行政处罚法(2021修订)](https://www.pkulaw.com/chl/6add260f579ab541bdfb.html?way=textRightFblx)

[中华人民共和国长江保护法](https://www.pkulaw.com/chl/adf90c0b432f8858bdfb.html?way=textRightFblx)

[中华人民共和国刑法(2020修正)](https://www.pkulaw.com/chl/39c1b78830b970eabdfb.html?way=textRightFblx)

[中华人民共和国民法典](https://www.pkulaw.com/chl/aa00daaeb5a4fe4ebdfb.html?way=textRightFblx)

[中华人民共和国固体废物污染环境防治法(2020修订)](https://www.pkulaw.com/chl/38e38019d3cd06adbdfb.html?way=textRightFblx)

[全国人民代表大会常务委员会关于废止有关收容教育法律规定和制度的决定](https://www.pkulaw.com/chl/1ea53f89482c77b7bdfb.html?way=textRightFblx)

[法规、司法解释备案审查工作办法](https://www.pkulaw.com/chl/fe86b1479330e044bdfb.html?way=textRightFblx)

[更多](https://www.pkulaw.com/AssociationMore/chl/6a190354a01feb78bdfb/2_100.html?way=textRightFblx)

©北大法宝：（[www.pkulaw.com](https://www.pkulaw.com/)）专业提供法律信息、法学知识和法律软件领域各类解决方案。北大法宝为您提供丰富的参考资料，正式引用法规条文时请与标准文本核对。 欢迎查看所有[产品和服务](http://www.pkulaw.net/)。
[法宝快讯： 如何快速找到您需要的检索结果？ 法宝 V6 有何新特色？](http://www.pkulaw.com/helps/69.html)

扫描二维码阅读原文

原文链接：<https://www.pkulaw.com/chl/6a190354a01feb78bdfb.html>
